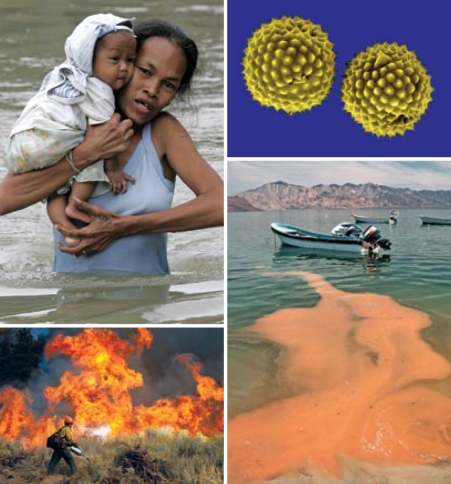# Sentinel Symptoms of Climate Change: Indicators for Related Health Effects

**DOI:** 10.1289/ehp.117-a504a

**Published:** 2009-11

**Authors:** Bob Weinhold

**Affiliations:** **Bob Weinhold**, MA, has covered environmental health issues for numerous outlets since 1996. He is a member of the Society of Environmental Journalists

Greenhouse gas emissions are widely acknowledged to be contributing to climate change–related health effects that vary by location, and are expected to continue doing so for many years, even if substantial emission cuts occur. A workgroup of the Council of State and Territorial Epidemiologists has identified a set of indicators that it says will allow national and local officials in the United States to better predict any such changes and consequences and to take appropriate action as it becomes warranted **[*EHP*** **117:1673–1681; English et al.]**. The team also identified the data needed for tracking these indicators and ascertained whether the data exist, must be improved, or must be generated. They say this is the first effort to synthesize and evaluate related information published by many sources.

The team determined the best indicators of environmental changes due to climate change are quantity of greenhouse gas emissions, air quality (in particular ozone), air mass stagnation events (such as those caused by temperature inversions), temperature and humidity, pollen loads, ragweed occurrence, drought incidence, drinking water scarcity, and occurrence of wildfires and harmful algal blooms. Data for some of these indicators are strong and/or expected to improve soon, as in the case of greenhouse gases, temperature, air mass stagnation events, and drought. Data on other indicators, such as pollen, harmful algal blooms, and ozone, require substantial improvement.

For indicators of human death and illness, the authors recommend tracking excess numbers of each that can be attributed to events related to climate change. Doing so will require significant improvements in existing data and methods, such as more comprehensive reporting of emergency room visits and hospitalizations related to heat waves, floods, and other extreme weather events. For infectious diseases, the targeted culprits are West Nile virus, Lyme disease, dengue fever, coccidioidomycosis, and hantavirus cardio pulmonary syndrome.

The authors note that some segments of the population may be especially vulnerable to certain effects of climate change. These groups include children; the elderly; pregnant and nursing women; those with disabilities and preexisting conditions such as asthma, chronic obstructive pulmonary disease, and obesity; people living in poverty or social isolation or without access to transportation; and those living within 5 km of a coast that is highly vulnerable to sea level rise, or in a 100- or 500-year flood zone. Awareness of these vulnerable subpopulations will be important in planning appropriate prevention and intervention activities.

Data for indicators of adaptability are sparse because most efforts so far have focused on mitigating climate change, not adapting to it. The authors propose that such indicators might include access to public cooling centers during heat waves, the existence of early warning systems for heat waves, mitigation plans to reduce urban heat islands, the number and quality of surveillance systems available to collect data on climate–health effects, and the availability of local public health workers and task forces trained in climate change research, surveillance, and adaptation.

## Figures and Tables

**Figure:**